# Whole genome assembly and annotation of the endangered Caribbean coral *Acropora cervicornis*

**DOI:** 10.1093/g3journal/jkad232

**Published:** 2023-10-06

**Authors:** Jason D Selwyn, Steven V Vollmer

**Affiliations:** Department of Marine and Environmental Sciences, Northeastern University, Nahant, MA 01908, USA; Department of Marine and Environmental Sciences, Northeastern University, Nahant, MA 01908, USA

**Keywords:** *Acropora cervicornis*, acroporidae, scleractinia, de novo assembly, comparative genomics, time-calibrated phylogeny

## Abstract

Coral species in the genus *Acropora* are key ecological components of coral reefs worldwide and represent the most diverse genus of scleractinian corals. While key species of Indo-Pacific *Acropora* have annotated genomes, no annotated genome has been published for either of the two species of Caribbean *Acropora*. Here we present the first fully annotated genome of the endangered Caribbean staghorn coral, *Acropora cervicornis*. We assembled and annotated this genome using high-fidelity nanopore long-read sequencing with gene annotations validated with mRNA sequencing. The assembled genome size is 318 Mb, with 28,059 validated genes. Comparative genomic analyses with other *Acropora* revealed unique features in *A. cervicornis*, including contractions in immune pathways and expansions in signaling pathways. Phylogenetic analysis confirms previous findings showing that *A. cervicornis* diverged from Indo-Pacific relatives around 41 million years ago, with the closure of the western Tethys Sea, prior to the primary radiation of Indo-Pacific *Acropora*. This new *A. cervicornis* genome enriches our understanding of the speciose *Acropora* and addresses evolutionary inquiries concerning speciation and hybridization in this diverse clade.

## Introduction

The *Acropora* are one of the most speciose and important genera of reef-building scleractinian corals globally (Wallace 1999). The genus *Acropora* are divided into multiple speciose Indo-Pacific clades and a single depauperate Caribbean clade (Wallace 1999). The two sister species of Caribbean *Acropora*—the Staghorn coral *A. cervicornis* and Elkhorn coral *A. palmata*—and their hybrid—called *A. prolifera* (Vollmer and Palumbi 2002) are thought to have diverged from the Indo-Pacific *Acropora* during the late Eocene after the closure of the western Tethys Sea prior to the rapid diversification in the Indo-Pacific *Acropora* (Wallace 1999; Wallace and Portell 2022). To date, all 16 published de novo assembled and annotated *Acropora* genomes are of Indo-Pacific species (Shinzato et al. 2011, Ying et al. 2019; Fuller et al. 2020; Shinzato et al. 2021; López-Nandam et al. 2023).

*Acropora*, like all corals, are severely threatened by anthropogenic climate change leading to elevated water temperatures that can cause acute bleaching and subsequently death (Hughes et al. 2018). The Caribbean *Acropora* are also experiencing a secondary range-wide pressure in the form of White Band Disease, which has resulted in ∼95% population losses of both species Caribbean wide and is the direct cause of their listing on the Endangered Species List (Aronson and Precht 2001; National Marine Fisheries Service 2006). Because these two species are such important foundational species in the Caribbean reef ecosystem, these losses have likely had tremendous unknown effects on higher order taxa which depend on *Acropora* dominant reefs for survival.

Here we present the first fully annotated genome for the endangered Caribbean staghorn coral *Acropora cervicornis*, importantly representing the first Caribbean species of this diverse clade. This genome was assembled using a combination of long-read nanopore and short-read shotgun sequences and annotated and validated using mRNA sequencing. This reference genome will accelerate genomic research on this endangered coral and address fundamental evolutionary questions about speciation and hybridization in the speciose Acroporids.

## Materials and methods

### Sample collection and sequencing

High molecular weight genomic DNA was extracted in June 2021 from adult tissue of the K2 genotype maintained in the Coral Restoration Foundation (CRF) Key Largo, Florida nursery. Three libraries were prepared using Oxford Nanopore Technologies (ONT) kit SQK-LSK112. Two libraries were not size selected while the third included 20+kb PippenPrep size-selection. All ONT prepared libraries were sequenced separately on three Minion flow cells (FLO-MIN112). High-quality base-calling was performed using Guppy v6.1.7 (ONT).

Four additional Illumina PCR-free shotgun libraries were constructed using the Discovar protocol to produce libraries with fragments between 400 and 600 bp (Love et al. 2016). KAPA PCR-free library kits were leveraged with the addition of a second round of 0.7× Agencourt AmPure XP SPRI bead cleanup post-adapter ligation. Libraries were multiplexed and sequenced on a single rapid-run HiSeq 2500 flowcell with 250 bp paired-end sequencing. Additionally, a library of paired 150 bp reads was prepared using the Illumina DNA Prep kit and sequenced as part of a NovaSeq S4 run.

To acquire transcriptome data, mRNA sequence data was obtained for 48 individuals (including the K2 genotype) using NEBs unidirectional mRNA library preparations sequenced on an Illumina NEXTSEQ 550 platform and combined with previously published RNA sequencing data from 38 additional *A. cervicornis* (PRJNA222758: Libro and Vollmer 2016; PRJNA423227: Parkinson et al. 2018).

### Sequence quality control

Nanopore long-reads (DNA) were quality controlled using Porechop (v 0.2.3_seqan2.1.1, https://github.com/rrwick/Porechop) to remove adapter sequences and then quality trimmed into longer assembly reads (minimum average quality 3, minimum length 1,000 bp) and shorter polishing reads (minimum average quality 5, minimum length 500 bp) using NanoFilt (De Coster et al. 2018). Illumina sequenced short-reads (DNA and RNA) were quality controlled initially using Fastp (Chen et al. 2018) to remove adapters and barcodes, filter low quality sequences (PHRED < 30), trim sequences shorter than 140 bp, and PCR artifacts. Contaminants were removed with Fastq_screen (Wingett and Andrews 2018) by mapping reads against a suite of potential contaminant genomes (e.g. human, viral, and bacterial) as well the 13 available genomes of *Symbiodiniaceae* (Supplementary Table 1) and removing reads which had hits to any potential contaminant genome.

### Genome assembly

We estimated the genome size using a k-mer counting approach implemented in Jellyfish based on the quality-controlled Illumina short reads (Marçais and Kingsford 2011). An initial genome assembly of the ONT sequenced reads was built using Flye (Kolmogorov et al. 2019) with the nano-hq parameter setting to fit with the chemistry and base-calling method of the sequencing. After initial assembly of the raw nanopore reads, duplicated sequences were removed using purge_dups (Guan et al. 2020; Guiglielmoni et al. 2021). We polished the genome using two rounds of long-read polishing with Racon (Vaser et al. 2017) followed by a round of polishing with Medaka v1.7.2 (ONT) and then two rounds of polishing with paired-end Illumina sequences using Pilon (Walker et al. 2014). To ensure a final genome assembly of the highest quality and contiguity, we corrected misassembly errors with short reads using Mec (Wu et al. 2020) and remove the mitochondrial genome and other potential genomic contaminants using Blobtools (Laetsch and Blaxter 2017). This was followed by misassembly correction using the long-read data (Coombe et al. 2021), scaffolding (Coombe et al. 2021), gap closing (Xu et al. 2020), and a final round of long-read polishing with Racon and short-read polishing with Pilon (Walker et al. 2014; Vaser et al. 2017). Finally, all contigs shorter than 1,000 bp and/or with no gene annotations (see below) were removed as they did not contain any BUSCOs.

### Transcriptome assembly

A preliminary transcriptome was assembled using a genome-guided assembly in Trinity (Grabherr et al. 2011). mRNA sequencing reads were splice-aware mapped to the genome using Gsnap (Wu et al. 2016). Trinity was used to perform a genome guided assembly of the transcriptome with an assumed max intron length of 100,000 bp.

### Genome annotation

Genome annotation was performed using MAKER (Cantarel et al. 2008; Holt and Yandell 2011). Repetitive elements were identified and masked using RepeatModeler (Flynn et al. 2020) and RepeatMasker (Smit et al. 2015). Evidence-based gene annotation was performed using the assembled transcriptome and proteins identified in either all Acroporids in the UniProt database (The UniProt Consortium 2023) or the closest reference proteomes in UniRef from *Stylophora pistillata* (Voolstra et al. 2017), *Pocillopora damicornis* (Cunning et al. 2018), *Actinia tenebrosa* (Surm et al. 2019), and *Nematostella vectensis* (Putnam et al. 2007). This initial round of annotation was used to train the ab initio gene identification models of Augustus (Stanke et al. 2006), Snap (Korf 2004), and Genemark-ES (Lomsadze et al. 2005). After the initial round of annotation based solely on protein and RNA evidence, we performed four subsequent rounds of annotation with the results of the previous round being used to train the ab initio gene predictors run in the subsequent round of annotation. Genes were functionally annotated using EnTAP (Hart et al. 2020) and Interproscan (Zdobnov and Apweiler 2001), and formatted the annotations for NCBI using GAG and Annie (Tate et al. 2014; Geib et al. 2018).

### Mitochondrial assembly

The mitochondrial genome was assembled using the quality-controlled Illumina short-reads using MitoZ (Meng et al. 2019). Briefly, this was done by first assembling a subset of reads into initial contigs which are then identified as mitogenome sequences using a profile Hidden Markov Model (Wheeler and Eddy 2013; Xie et al. 2014; Nurk et al. 2017). Contigs were then annotated to find the 13 protein-coding mitochondrial genes along with tRNAs and rRNAs with any contigs not containing any annotations removed (Birney et al. 2004; Gertz et al. 2006; Li and Durbin 2009; Jühling et al. 2012; Nawrocki and Eddy 2013). Finally, retained contigs were assembled and circularized into the complete mitochondrial genome and visualized (Gertz et al. 2006; Krzywinski et al. 2009; Meng et al. 2019).

### Phylogenetic analysis

Sixteen published *Acropora* genomes with structural genome annotations along with two *Montipora* species, *Montipora capitata* and *Montipora efflorescens*; two Pocilloporids, *P. damicornis* and *S. pistillata*; and three anemones, *N. vectensis*, *A. tenebrosa*, and *Exaiptasia diaphana* were downloaded from NCBI (Table 1). Protein sequences for all annotated genes were extracted and clustered into orthogroups derived from a single gene in the last common ancestor of the group using OrthoFinder (Emms and Kelly 2019). Orthogroups were used to infer rooted gene and species trees to develop a phylogenetic hypothesis for the group (Emms and Kelly 2017, 2018). Specifically, we used the STAG (Emms and Kelly 2018) algorithm to infer the species tree from 7,110 multicopy gene trees which had all species present, each created using DendroBLAST (Kelly and Maini 2013), this species tree was then rooted using the STRIDE algorithm (Emms and Kelly 2017). To infer divergence times, we time-calibrated the species tree using least-squares dating and 1,000 bootstraps to estimate divergence time confidence intervals (To et al. 2016). Ancestral dates were gathered from the Fossilworks database (Supplementary Table 2; Behrensmeyer and Turner 2013).

**Table 1.** National Center for Biotechnology Information accession numbers and citations for coral genomes with structural gene annotations used to build phylogeny and for comparative genomic analysis.

Species	Genome size (Mb)	Number of genes	Accession number	Citation
*Acropora acuminata*	394.7	26,151	GCA_014633975	Shinzato et al. (2021)
*Acropora awi*	428.8	26,801	GCA_014634005	Shinzato et al. (2021)
*Acropora cervicornis*	308	28,059	GCA_032359415	(This Study)
*Acropora cytherea*	426.3	27,327	GCA_014634045	Shinzato et al. (2021)
*Acropora digitifera*	415.8	25,278	GCA_014634065	Shinzato et al. (2021)
*Acropora echinata*	401.5	26,170	GCA_014634105	Shinzato et al. (2021)
*Acropora florida*	442.8	27,573	GCA_014634605	Shinzato et al. (2021)
*Acropora gemmifera*	401	26,269	GCA_014634125	Shinzato et al. (2021)
*Acropora hyacinthus*	447.2	27,215	GCA_014634145	Shinzato et al. (2021)
*Acropora intermedia*	416.9	26,982	GCA_014634585	Shinzato et al. (2021)
*Acropora microphthalma*	383.9	26,384	GCA_014634165	Shinzato et al. (2021)
*Acropora millepora*	475.4	41,860	GCA_013753865	Fuller et al. (2020)
*Acropora muricata*	420.7	27,409	GCA_014634545	Shinzato et al. (2021)
*Acropora nasuta*	416.4	27,379	GCA_014634205	Shinzato et al. (2021)
*Acropora selago*	392.9	27,036	GCA_014634525	Shinzato et al. (2021)
*Acropora tenuis*	403.1	27,236	GCA_014633955	Shinzato et al. (2021)
*Acropora yongei*	438	27,452	GCA_014634225	Shinzato et al. (2021)
*Montipora cactus*	652.7	29,158	GCA_014634245	Shinzato et al. (2021)
*Montipora efflorescens*	643.3	29,424	GCA_014634505	Shinzato et al. (2021)
*Nematostella vectensis*	356.6	34,311	GCF_000209225	Putnam et al. (2007)
*Pocillopora damicornis*	234.3	25,183	GCF_003704095	Cunning et al. (2018)
*Stylophora pistillata*	397.6	33,252	GCF_002571385	Voolstra et al. (2017)
*Actinia tenebrosa*	238.2	27,037	GCF_009602425	Surm et al. (2019)
*Exaiptasia diaphana*	256.1	27,753	GCF_001417965	Baumgarten et al. (2015)

### Comparative genomics

Structural gene annotations for all species included in the phylogenetic hypothesis were functionally annotated using BLAST (Altschul et al. 1990; Camacho et al. 2009) against the Swiss-Prot curated portion of the UniProt database (The UniProt Consortium 2023). To identify KEGG orthologs for all orthogroups, we matched KEGG gene annotations to orthogroups across species and found the consensus KEGG ortholog across all species in which the orthogroup was found. KEGG ortholog membership within KEGG pathways was identified using KEGGREST (Tenenbaum and Volkening 2023).

We used a logistic regression model to test if there are differences in the percentage of orthogroups with KEGG annotations across taxa. Post-hoc tests were used to compare the rate of annotation in *A. cervicornis* to other *Acropora* sp. and make pairwise comparisons between genera. To identify systematic differences in the distribution of genes in pathways within the *Acropora*, we used *χ*^2^ tests followed by a post-hoc analysis with FDR correction to identify the species and KEGG pathways with significantly more or less gene copies (i.e. orthogroups) than would be expected compared to the other *Acropora* (Beasley and Schumacker 1995; Benjamini and Hochberg 1995).

The time-calibrated phylogeny along with the number of genes found within orthogroups for each species was used to estimate the rate of gene family evolution across the phylogeny using Cafe5 (Hahn et al. 2005; Mendes et al. 2020) and identify KEGG orthologs which exhibit significant expansions/contractions at each node of the phylogeny. In the Cafe5 analysis we assumed a single rate of evolution across all gene families using the error rate estimating model and Poisson prior distribution (De Bie et al. 2006; Han et al. 2013). We then performed an overrepresentation analysis using Fisher's exact test to determine if any pathways in *A. cervicornis* showed significant expansions/contractions. All statistical analyses were performed using R v4.2.1 (R Core Team 2022).

## Results and discussion

### Genome/transcriptome assembly and annotation

Nanopore sequencing resulted in ∼6.6 million reads containing 15.5 Gb with an N50 of 5,072 bp with 3.3 million high-quality reads after filtering used in the initial long-read assembly containing 13.3 Gb of DNA with an N50 of 6,078 bp and a polishing set of 4.5 million reads containing 13.3 Gb of DNA with an N50 of 5,269 bp. 92 million paired-end Illumina reads (totally 40 Gb) were retained after filtering and decontamination for *x* polishing. mRNA sequencing from 86 corals totaled 1.4 billion single-end reads and 174 Gb of mRNA sequencing data for gene annotation.

**Fig. 1.**
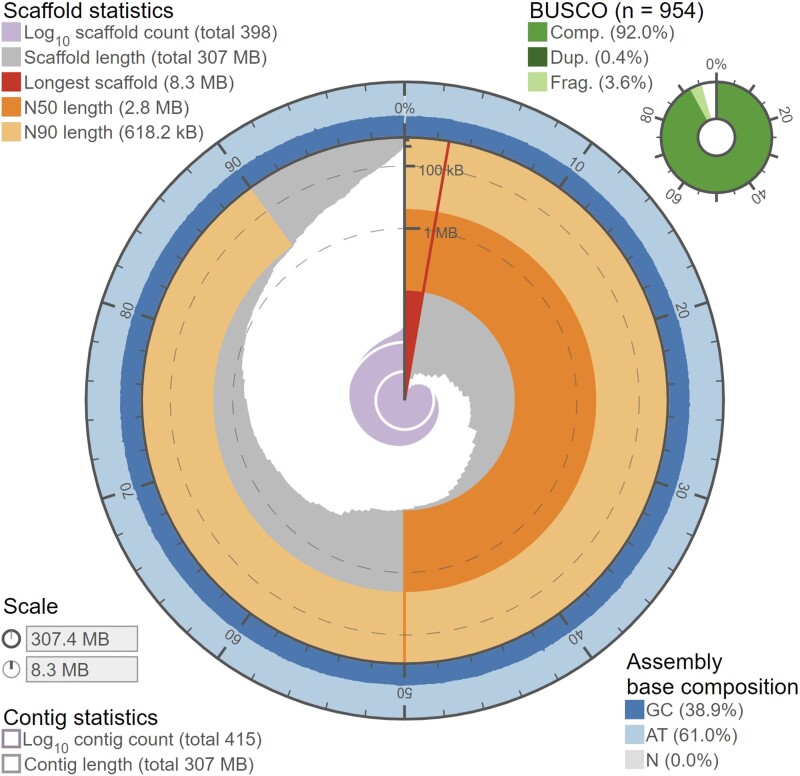
Genome snail plot showing genome contiguity and completeness statistics. For an interactive version of the figure see: https://jdselwyn.github.io/assembly-stats/. Created using Challis (2017).

Using the filtered paired-end short-read sequences, we estimated the genome size of *A. cervicornis* to be ∼318 Mb, somewhat smaller than the Pacific Acroporids (384–475 Mb; Shinzato et al. 2011, 2021; Ying et al. 2019; Fuller et al. 2020), and obtained ∼42× genomic coverage of the long-read nanopore sequences for the genomic assembly. The initial genome assembly measured 328 Mb with 3,014 contigs (N50 = 0.94 Mb; L50 = 99) with the longest contig being 4.9 Mb and a BUSCO completeness of 92.8% (1.2% duplicated, 3.7% fragmented). After genomic post-processing, polishing, and scaffolding, the final genome assembly measured 307 Mb (96.5% the estimated genome size, Table 2) with 398 scaffolded contigs (N50 = 2.8 Mb; L50 = 35) with the longest scaffold measuring 8.3 Mb and a BUSCO completeness of 92.4% (0.4% duplicated, 3.6% fragmented, Fig. 1). The pooled transcriptome contained 374,749 transcripts with a total N50 of 3,659 and longest isoform N50 of 1,483 with a BUSCO completeness of 93.4% (78% duplicated, 3.5% fragmented). The high degree of transcriptome duplication in the BUSCO value is likely due to the pooling of individuals to create the transcriptome. Similar to other *Acropora* genomes (Shinzato et al. 2021), *A. cervicornis* contained 39% interspersed repeats (Supplementary Table 3) with the most common identifiable class of repeat being short interspersed nuclear elements, though the plurality (17%) of repeats in the genome were unable to be classified and may be taxon specific (Shinzato et al. 2021). *A. cervicornis* had 28,059 validated genes (Table 3), intermediate among other Acroporids (25,278–41,860; Shinzato et al. 2011; Ying et al. 2019; Fuller et al. 2020; Shinzato et al. 2021), with 53% having a Swiss-Prot annotation at an *e*-value < 10^−6^ and a BUSCO completeness of 81.6% (2.8% duplicated, 10% fragmented).

**Table 2.** *A. cervicornis* assembly statistics.

Cumulative scaffold length (bp)	307.4
Number of scaffolds	398
Number of contigs	415
G + C content (%)	38.95
Number of Ns/100 kbp	37.23
Largest scaffold (Mb)	8.337
Scaffold N50 (Mb)	2.8
Scaffold L50	35
Largest contig (Mb)	8.337
Contig N50 (Mb)	2.7
Contig L50 (Mb)	36
BUSCO complete single-copy	878
BUSCO complete multicopy	4
BUSCO fragmented	34
BUSCO missing	38

**Table 3.** *A. cervicornis* annotation statistics.

Number of genes	28,059
Cumulative length of CDSs (bp)	36,143,406
Median gene length (bp)	4,072
Median CDS length (bp)	119
Median exon length (bp)	124
Median intron length (bp)	569
Number of intronless genes	4,591
Median number of exons per gene	4
Median number of exons per multiexon gene	4
BUSCO complete single-copy	752
BUSCO complete multicopy	27
BUSCO fragmented	95
BUSCO missing	80

### Mitochondrial genome

**Fig. 2.**
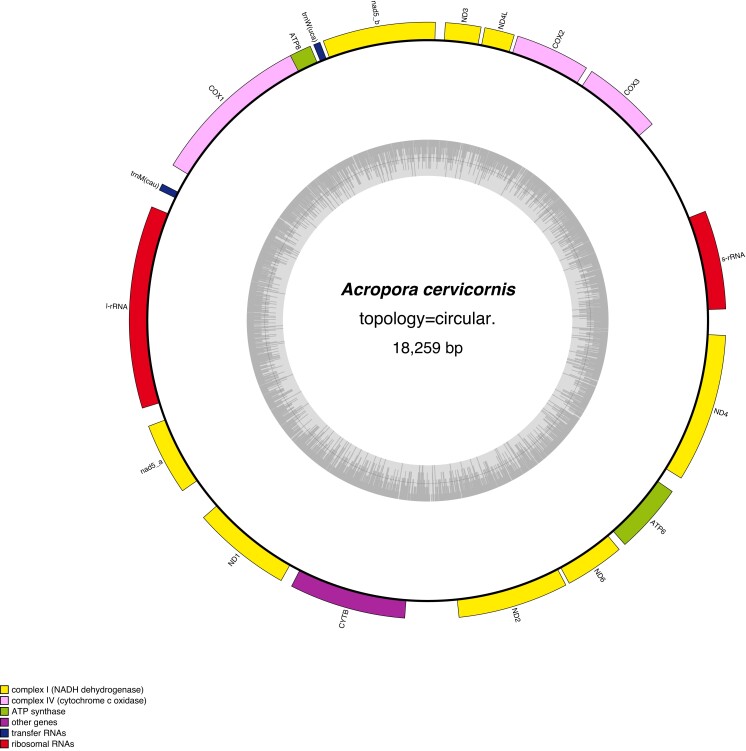
Mitochondrial genome plot showing the relative locations and orientations of genes on the mitochondria with gene groups color coded by type. Plot made using Lohse et al. 2013).

The *A. cervicornis* mitochondrial genome was assembled into a single circular structure containing 18,259 bp, 13 protein-coding genes, two tRNA genes (tRNA-Met and tRNA-Trp), and two rRNA genes (16 and 12 s; Fig. 2, GenBank accession number: OQ772303). Like all *Acropora,* the *A. cervicornis* mitochondrial genome has a particular dearth of tRNA coding sequences when compared to other metazoans, likely due to this diversification occurring after the split between cnidarians and metazoans (van Oppen et al. 1999; van Oppen, Catmull, *et al*. 2002; Liu et al. 2015; Tian and Niu 2017; Colin et al. 2021). The gene order found in *A. cervicornis* is the same as that found in other Acroporids, including ND5 being split into two portions with a large intron containing all genes except the two tRNA coding genes, the large ribosomal subunit rRNA, ATP8, and COI (Figure Y; van Oppen, Catmull, *et al*. 2002; Liu et al. 2015; Zhang et al. 2016; Colin et al. 2021).

### Comparative genomics

The 28,059 genes found in *A. cervicornis* belong to 15,191 distinct orthogroups of which 54.4% had a KEGG annotation. The percent of orthogroups with KEGG annotations varied significantly by species (*χ*^2^_(23)_ = 595.8, *P* < 0.0001) due primarily to differences between genera (*Acropora* vs *Montipora*, *Z* = 12.9, *P* < 0.0001) and higher-order taxonomic comparisons outside of the *Acropora* (*Acropora* vs outgroups, *Z* = −17.6, *P* < 0.0001) rather than within *Acropora* which all show a similar degree of annotation (*A. cervicornis* vs other *Acropora*, Z = −0.87, *P* = 0.38). The number of orthogroups per KEGG pathway were similar for all *Acropora* sp. (*χ*^2^_(4,976)_ = 478.1, *P* = 1) indicating that no Acropora genomes had broad gains or losses of genes. Amongst five major KEGG categories, 25.7% of the orthogroups were found in pathways involved in organismal systems and 24.8% were involved in environmental information processing. The remaining orthogroups were split between cellular processes (18.8%), metabolism-related processes (16.7%), and genetic information processing (14.1%). *A. cervicornis* possessed 135 unique orthogroups, more than the other *Acropora* species (17–70), possibly as a result of the separate evolutionary history in the Caribbean. An alternative hypothesis for this discrepancy could be that the ab initio gene models for most other *Acropora* were trained on *A. digitifera* rather than being trained for each species independently (Shinzato et al. 2021).

Six gene pathways were significantly overrepresented in *A. cervicornis* among the significantly expanded and contracted KEGG orthologs (Supplementary Table 4). Two immune system pathways—NOD-like receptor signaling (map04621, OR = 19.7, *P* < 0.001, *p_adj_* < 0.001) and Neutrophil extracellular trap formation (map04613, OR = 16.9, *P* < 0.001, *p_adj_* = 0.018)—and the related cellular growth and death pathway Necroptosis (map04217, OR = 17.6, *P* < 0.001, *p_adj_* = 0.018) were significantly overrepresented due to one expansion and three contractions in NACHT, LRR, and PYD domain-containing proteins, three contractions in histone proteins (H2A, H3, and H4), and one expansion in a cation channel protein (TRPM7). In corals, NOD-like receptor signaling pathway has been found to be an integral part of the innate immune system (Hamada et al. 2013) and histone phosphorylation has been shown to be a key response to nutrient stress and regulating the dinoflagellate symbionts (Rodriguez-Casariego et al. 2018).

The second major group of overrepresented pathways are signaling pathways, particularly the calcium signaling pathway (map04020, OR = 9.93, *P* < 0.001, *p_adj_* = 0.025) and the neuroactive ligand–receptor interaction (map04080, OR = 39.8, *P* < 0.001, *p_adj_* < 0.001). While the immune-related pathways were predominantly gene contractions in these pathways, all KEGG orthologs showed expansions (Supplementary Table 4). The calcium signaling and neuroactive ligand–receptor pathways have been found to be important in regulation reproduction (Hilton et al. 2012; Rosenberg et al. 2017), nematocyst regulation (Russell and Watson 1995), and biomineralization (Reyes-Bermudez et al. 2009). Further, these pathways appear to be differentially expressed between the growth tips and bases in the Caribbean *Acropora* (Hemond et al. 2014).

The final KEGG pathway overrepresented among the rapidly evolving KEGG orthologs is the Taurine and hypotaurine metabolism pathway (map00430, OR = 91.6, *P* < 0.001, *p_adj_* = 0.04). This pathway was driven by the loss of a single KEGG orthogroup, cysteine dioxygenase (−2, K00456). Unlike other scleractinian corals, Indo-Pacific *Acropora* lack the cystathionine beta-synthase gene involved in cysteine biosynthesis (Shinzato et al. 2011) suggesting that *Acroporid* corals must rely on their algal symbionts to supply this vital amino acid (Shinzato et al. 2014). However, an alternative cysteine biosynthesis pathway has recently been discovered in *Acropora loripes* suggesting *Acropora* corals can natively synthesize cysteine through this alternate pathway (Salazar et al. 2022). Similar to the other studied *Acropora*, *A. cervicornis* lacks genes coding for cystathionine beta-synthase but does possess the genes required for the alternate cysteine biosynthesis pathway (Salazar et al. 2022). The loss of cysteine dioxygenase in *A. cervicornis* may suggest that the alternative pathway does not fully compensate the biosynthesis of cysteine resulting in a lack of excess cysteine needing to be metabolized.

### Phylogeny

**Fig. 3.**
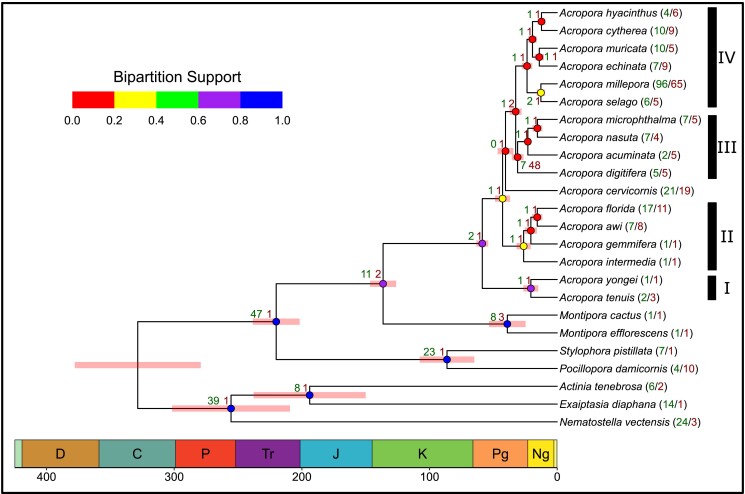
Species phylogeny based on identified orthogroups. Node color indicates the proportion of single-locus gene trees supporting each bipartition (i.e. gene concordance factor, a more stringent metric of node support than bootstrap support, Emms and Kelly 2018; Minh et al. 2020). Red bars at each node indicate the 95% confidence interval of the estimated time of divergence marked in geologic periods on the *x* axis. Green/red node numbers indicate the number of KEGG orthologs which significantly expanded/contracted at each divergence point. Clade groupings from Shizato et al. (2021).

*A. cervicornis* is estimated to have diverged from the other *Acropora* 41 million years ago (mya) (35–47, 95% CI) during the Paleogene, approximately coinciding with the initial closure of the Tethys Sea (van Oppen et al. 2001; Wallace and Portell 2022). The addition of *A. cervicornis* as a Caribbean *Acropora* to the published *Acropora* phylogenomic tree (Shinzato et al. 2021) suggests that the *Acropora* radiation in the Indo-Pacific occurred in at least two stages with an initial split occurring between 58 and 68 mya resulting in clades I and II prior to the divergence of the Caribbean and Indo-Pacific *Acropora*, followed by a second set of radiations of the Indo-Pacific *Acropora* 35–47 mya and resulting in the more speciose clades III and IV (Fig. 3). Low bipartition support values within *Acropora* could result from the relatively rapid radiation within *Acropora*, incomplete lineage sorting and/or introgressive hybridization (Vollmer and Palumbi 2002; van Oppen, Willis, et al. 2002; Minh et al. 2020). The Acroporids (*Montipora* and *Acropora*) likely diverged from the Pocilloporids (*S. pistillata* and *P. damicornis*) during the Triassic period 220 mya (188–225, 95% CI) with *Acropora* and *Montipora* diverging during the early Cretaceous 136 mya (116–136, 95% CI).

### Conclusion

In summary, this study presents the first fully annotated genome of the endangered Caribbean staghorn coral, *A. cervicornis*. Comparative genomics highlights distinctive genetic traits, including immune pathway contractions and signaling pathway expansions, suggesting further research into the species response to environmental stressors, particularly White Band Disease. Phylogenetic analysis places *A. cervicornis* within the broader *Acropora* genus, dating its divergence to around 41 mya, aligning previous morphological findings and supporting the hypothesis of divergence coincident with the closure of the western Tethys Sea. This annotated genome serves as a valuable resource for future research, facilitating conservation and restoration efforts for Caribbean coral reefs, and deepening our understanding of speciation and adaptation within *Acropora*.

## Supplementary Material

jkad232_Supplementary_Data

## Data Availability

Genome assembly and associated Nanopore and short-read DNA sequencing data can be accessed from NCBI BioProject: PRJNA948411 with the mitochondrial genome assembly accessible at NCBI GenBank: OQ772303. RNA sequencing data used for annotation can be accessed from NCBI BioProject: PRJNA949884. Genome assembly and annotation pipeline code available from GitHub: https://github.com/VollmerLab/Acerv_Genome. Supplemental material available at G3 online.
