# Supplementary material for: Whole genome assembly and annotation of the endangered Caribbean coral Acropora cervicornis
Source: G3 (Bethesda). 2023 Oct 6;13(12):jkad232. doi: 10.1093/g3journal/jkad232 (PMC10700113; doi:10.1093/g3journal/jkad232)
Supplement: jkad232_Supplementary_Data [file jkad232_supplementary_data.docx]

**Supplemental Tables**

Table S1: National Center for Biotechnology Information accession numbers and citations for Symbiodinium reference genomes which reads were mapped against to identify Symbiodinium composition within each sample. Species names have been adjusted sensu LaJeunesse et al. (2018).

| Species | Clade | Accession # | Citation |
| --- | --- | --- | --- |
| *Symbiodinium microadriaticum* | A | GCA_001939145 | (Aranda *et al.* 2016) |
| *Symbiodinium sp.* clade A Y106 | A | GCA_003297005 | (Shoguchi *et al.* 2018) |
| *Cladocopium sp.* clade C Y103 | C | GCA_003297045 | (Shoguchi *et al.* 2018) |
| *Fugacium kawagutii* | F | GCA_009767595 | (Lin *et al.* 2015) |
| *Symbiodinium microadriaticum* | A | GCA_018327485 | (Yoshioka *et al.* 2021) |
| *Symbiodinium natans* | A | GCA_905221605 | (González-Pech *et al.* 2021) |
| *Symbiodinium sp.* CCMP2592 | A | GCA_905221615 | (González-Pech *et al.* 2021) |
| *Symbiodinium sp.* KB8 | A | GCA_905221625 | (González-Pech *et al.* 2021) |
| *Symbiodinium sp.* CCMP2456 | A | GCA_905221635 | (González-Pech *et al.* 2021) |
| *Symbiodinium pilosum* | A | GCA_905231905 | (González-Pech *et al.* 2021) |
| *Symbiodinium necroappetens* | A | GCA_905231915 | (González-Pech *et al.* 2021) |
| *Symbiodinium microadriaticum* | A | GCA_905231925 | (González-Pech *et al.* 2021) |
| *Breviolum minutum* Mf 1.05b.01 | B | GCA_000507305 | (Shoguchi *et al.* 2013) |

Table S2: Fossil calibration timepoints. Clade indicates the group of extant species whose most recent common ancestor is estimated based on fossil evidence between Lower and Upper

| Clade | Fossil Calibration (mya) |
| --- | --- |
| *Pocillopora damicornis* & *Acropora* | 164.7 - 225.1 |
| *Montipora* & *Acropora* | 70.6 - 136.4 |
| *Montipora cactus* & *Montipora efflorescens* | 5.3 - 70.6 |
| *Acropora* | 58.7 - 70.6 |
| *Acropora intermedia* & *Acropora awi* | 20.4 - 58.7 |
| *Acropora microphthalma* & *Acropora acuminata* | 23.0 - 58.7 |
| *Acropora echinate* & *Acropora millepora* | 16.7 - 58.7 |

Table S3: Repetitive Elements

|  |  |  | # Elements | Length (bp) | % of sequence |
| --- | --- | --- | --- | --- | --- |
| Retroelements |  |  | 111,792 | 32,563,176 | 10.56 |
|  | SINEs: |  | 21,510 | 3,324,049 | 1.08 |
|  | Penelope |  | 37,570 | 8,352,404 | 2.71 |
|  | LINES: |  | 62,230 | 18,534,800 | 6.01 |
|  |  | CRE/SLACS | 1,342 | 490,978 | 0.16 |
|  |  | L2/CR1/Rex | 15,517 | 5,509,306 | 1.79 |
|  |  | R1/LOA/Jockey | 0 | 0 | 0 |
|  |  | R2/R4/NeSL | 2,784 | 1,152,809 | 0.37 |
|  |  | RTE/Bov-B | 1,161 | 557,089 | 0.18 |
|  |  | L1/CIN4 | 871 | 589,474 | 0.19 |
|  | LTR elements: |  | 28,052 | 10,704,327 | 3.47 |
|  |  | BEL/Pao | 3,365 | 1,885,400 | 0.61 |
|  |  | Ty1/Copia | 1,860 | 731,160 | 0.24 |
|  |  | Gypsy/DIRS1 | 13,894 | 6,078,170 | 1.97 |
|  |  | Retroviral | 404 | 129,000 | 0.04 |
| DNA transposons |  |  | 115,494 | 28,154,224 | 9.13 |
|  | hobo-Activator |  | 21,307 | 5,772,943 | 1.87 |
|  | Tc1-IS630-Pogo |  | 9,479 | 2,066,913 | 0.67 |
|  | En-Spm |  | 0 | 0 | 0 |
|  | MuDR-IS905 |  | 0 | 0 | 0 |
|  | PiggyBac |  | 395 | 244,277 | 0.08 |
|  | Tourist/Harbinger |  | 15,886 | 3,057,020 | 0.99 |
|  | Other (Mirage, P-element, Transib) |  | 1,166 | 403,603 | 0.13 |
| Rolling-circles |  |  | 2,807 | 809,721 | 0.26 |
| Unclassified |  |  | 261,702 | 53,601,606 | 17.38 |
| Total interspersed repeats |  |  |  | 114,319,006 | 37.08 |
| Small RNA |  |  | 10,386 | 1,603,476 | 0.52 |
| Satellites |  |  | 1,023 | 725,695 | 0.24 |
| Simple repeats |  |  | 56,201 | 2,585,746 | 0.84 |
| Low complexity |  |  | 8,032 | 381,669 | 0.12 |

Table S4: A. cervicornis KEGG gene expansions & contractions in pathways overrepresented among expanding/contracting orthogroups.

| Gene Name (KEGG ID) | Change | Pathway(s) |
| --- | --- | --- |
| NACHT, LRR and PYD domain-containing protein 1 (K12798) | -2 | NOD-like receptor signaling (map04621) |
| NACHT, LRR and PYD domain-containing protein 3 (K12800) | -2 | NOD-like receptor signaling (map04621); Necroptosis (map04217) |
| NACHT, LRR and PYD domain-containing protein 7 (K20864) | +2 | NOD-like receptor signaling (map04621) |
| NACHT, LRR and PYD domain-containing protein 12 (K20865) | -8 | NOD-like receptor signaling (map04621) |
| Histone H2A (K11251) | -2 | Neutrophil extracellular trap formation (map04613); Necroptosis (map04217) |
| Histone H3 (K11253) | -4 | Neutrophil extracellular trap formation (map04613) |
| Histone H4 (K11254) | -3 | Neutrophil extracellular trap formation (map04613) |
| TRPM7 - transient receptor potential cation channel subfamily M member 7 (K04982) | +4 | NOD-like receptor signaling (map04621); Necroptosis (map04217) |
| adrenergic receptor beta-2 (K04142) | +9 | Calcium signaling pathway (map04020); Neuroactive ligand-receptor interaction (map04080) |
| dopamine receptor D1 (K04144) | +4 | Calcium signaling pathway (map04020); Neuroactive ligand-receptor interaction (map04080) |
| tachykinin receptor 2 (K04223) | +14 | Calcium signaling pathway (map04020); Neuroactive ligand-receptor interaction (map04080) |
| adenosine receptor A2b (K04267) | +4 | Calcium signaling pathway (map04020); Neuroactive ligand-receptor interaction (map04080) |
| 5-hydroxytryptamine receptor 1 (K04153) | +3 | Neuroactive ligand-receptor interaction (map04080) |
| melanocortin 4 receptor (K04202) | +3 | Neuroactive ligand-receptor interaction (map04080) |
| melanocortin 5 receptor (K04203) | +4 | Neuroactive ligand-receptor interaction (map04080) |
| melatonin receptor type 1A (K04285) | +4 | Neuroactive ligand-receptor interaction (map04080) |
| trace amine associated receptor (K05051) | +18 | Neuroactive ligand-receptor interaction (map04080) |
| neuropeptide FF receptor 2 (K08375) | +8 | Neuroactive ligand-receptor interaction (map04080) |
| pyroglutamylated RFamide peptide receptor (K08378) | +3 | Neuroactive ligand-receptor interaction (map04080) |
| cysteine dioxygenase (K00456) | -2 | Taurine and hypotaurine metabolism (map00430) |

**Supplemental Literature Cited**

Aranda, M., Y. Li, Y. J. Liew, S. Baumgarten, O. Simakov *et al.*, 2016 Genomes of coral dinoflagellate symbionts highlight evolutionary adaptations conducive to a symbiotic lifestyle. Sci Rep 6: 39734.

González-Pech, R. A., T. G. Stephens, Y. Chen, A. R. Mohamed, Y. Cheng *et al.*, 2021 Comparison of 15 dinoflagellate genomes reveals extensive sequence and structural divergence in family Symbiodiniaceae and genus Symbiodinium. BMC Biol 19: 73.

LaJeunesse, T. C., J. E. Parkinson, P. W. Gabrielson, H. J. Jeong, J. D. Reimer *et al.*, 2018 Systematic Revision of Symbiodiniaceae Highlights the Antiquity and Diversity of Coral Endosymbionts. Current Biology 28: 2570-2580.e6.

Lin, S., S. Cheng, B. Song, X. Zhong, X. Lin *et al.*, 2015 The *Symbiodinium kawagutii* genome illuminates dinoflagellate gene expression and coral symbiosis. Science 350: 691–694.

Shoguchi, E., G. Beedessee, I. Tada, K. Hisata, T. Kawashima *et al.*, 2018 Two divergent *Symbiodinium* genomes reveal conservation of a gene cluster for sunscreen biosynthesis and recently lost genes. BMC Genomics 19: 458.

Shoguchi, E., C. Shinzato, T. Kawashima, F. Gyoja, S. Mungpakdee *et al.*, 2013 Draft assembly of the *Symbiodinium minutum* nuclear genome reveals dinoflagellate gene structure. Curr Biol 23: 1399–1408.

Yoshioka, Y., H. Yamashita, G. Suzuki, Y. Zayasu, I. Tada *et al.*, 2021 Whole-Genome Transcriptome Analyses of Native Symbionts Reveal Host Coral Genomic Novelties for Establishing Coral-Algae Symbioses. Genome Biol Evol 13: evaa240.
